# Supplementary material for: Aflatoxin B1 Contamination Association with the Seed Coat Biochemical Marker Polyphenol in Peanuts Under Intermittent Drought
Source: J Fungi (Basel). 2024 Dec 10;10(12):850. doi: 10.3390/jof10120850 (PMC11679287; doi:10.3390/jof10120850)
Supplement: Supplementary file 1 [file jof-10-00850-s001.zip › jof-3172296-supplementary.pdf]

Journal of *Fungi*

MDPI

Supplementary Table S1. Yield and its components under well-watered (WW) and water-stressed (WS) treatments and least significant differences at the 5% level (LSD). PNP: Pod number per plant; SNP: Seed number per plant; IMPN: Immature pod number per plant; HY: Haulm yield; PY: Pod yield; and SY: Seed yield.

	Well-Watered						Water-Stressed					
**Genotypes**	**PNP**	**SNP**	**IMPN**	**PY (gm^{-2})**	**SY (gm^{-2})**	**HY (gm^{-2})**	**PNP**	**SNP**	**IMPN**	**PY (gm^{-2})**	**SY (gm^{-2})**	**HY (gm^{-2})**
55-437	103 ± 2	124 ± 17	8 ± 2	594.7 ± 96	290.6 ± 49	736.4 ± 292	95 ± 11	109 ± 14	15 ± 1	517 ± 51	248.2 ± 46	612.8 ± 220
Fleur 11	99 ± 12	136 ± 13	9 ± 1	613.5 ± 81	338.6 ± 79	870.1 ± 75	89 ± 5	119 ± 16	17 ± 2	572 ± 138	316.2 ± 33	658 ± 160
ICG 10950	98 ± 5	112 ± 19	4 ± 1	701.6 ± 61	376.8 ± 70	1022.4 ± 154	79 ± 8	70 ± 14	16 ± 2	638.8 ± 30	284.3 ± 10	740.3 ± 97
ICG 11322	76 ± 22	72 ± 12	4 ± 1	567.8 ± 33	272.8 ± 39	691.6 ± 118	62 ± 14	65 ± 9	26 ± 2	529 ± 49	242.3 ± 31	583.9 ± 127
ICG 1142	112 ± 6	112 ± 8	12 ± 2	1664.9 ± 255	369.4 ± 78	798.6 ± 69	84 ± 10	78 ± 4	23 ± 2	1247.9 ± 283	328 ± 82	666.6 ± 151
ICG 12235	207 ± 16	116 ± 18	34 ± 3	766.6 ± 154	188.5 ± 32	930.1 ± 82	141 ± 6	56 ± 13	76 ± 3	677 ± 128	183.4 ± 17	756.6 ± 148
ICG 12697	112 ± 7	137 ± 5	9 ± 2	560.6 ± 76	395.4 ± 70	637.2 ± 245	75 ± 6	100 ± 9	17 ± 3	363.4 ± 50	245.3 ± 19	513.6 ± 85
ICG 12879	101 ± 2	129 ± 11	8 ± 2	472.2 ± 68	306.9 ± 27	584.7 ± 184	86 ± 6	64 ± 20	16 ± 2	351.7 ± 33	211.7 ± 48	536.9 ± 58
ICG 12988	101 ± 17	131 ± 21	10 ± 2	649.7 ± 148	280.9 ± 92	562.2 ± 80	76 ± 13	102 ± 11	17 ± 2	388 ± 38	206.5 ± 35	533.6 ± 86
ICG 12991	115 ± 8	153 ± 21	4 ± 1	525.3 ± 42	243.8 ± 36	657.5 ± 203	79 ± 10	86 ± 4	17 ± 3	443.9 ± 33	199.5 ± 25	629.5 ± 169
ICG 13099	111 ± 3	133 ± 9	8 ± 1	889.1 ± 235	370.8 ± 120	925.1 ± 86	88 ± 10	85 ± 20	21 ± 2	653.8 ± 98	286.3 ± 51	741.1 ± 133
ICG 13603	177 ± 12	205 ± 26	10 ± 1	780.9 ± 83	356.2 ± 111	775.4 ± 58	134 ± 9	163 ± 20	25 ± 1	662.5 ± 131	256.6 ± 17	589.9 ± 102
ICG 13858	76 ± 8	112 ± 14	8 ± 3	475.7 ± 80	196.0 ± 57	910.5 ± 48	48 ± 4	67 ± 14	21 ± 4	397.5 ± 20	134.8 ± 32	552.6 ± 159
ICG 14106	92 ± 8	142 ± 17	8 ± 2	833.5 ± 47	321.4 ± 35	724 ± 162	53 ± 8	74 ± 13	19 ± 2	776.9 ± 145	252.1 ± 50	513.4 ± 50
ICG 1415	100 ± 5	98 ± 11	13 ± 2	655.3 ± 78	282.7 ± 28	767.3 ± 76	86 ± 10	71 ± 12	22 ± 5	624.3 ± 67	209.6 ± 34	589.7 ± 135
ICG 14523	159 ± 13	174 ± 12	10 ± 2	906.0 ± 154	378.6 ± 31	954.3 ± 58	121 ± 12	111 ± 3	21 ± 3	746.5 ± 27	298.8 ± 25	780.2 ± 125
ICG 14630	53 ± 7	84 ± 10	9 ± 2	725.4 ± 107	206.9 ± 61	893.5 ± 224	50 ± 4	67 ± 8	15 ± 3	654.8 ± 68	150.6 ± 12	636.2 ± 262
ICG 1519	86 ± 5	79 ± 15	8 ± 2	590.5 ± 96	214 ± 55	761.5 ± 185	63 ± 11	70 ± 16	20 ± 3	485.6 ± 16	180.9 ± 36	695.4 ± 208
ICG 156	102 ± 14	125 ± 11	9 ± 1	682 ± 84	237.9 ± 24	1602 ± 468	84 ± 2	83 ± 9	21 ± 3	432.6 ± 58	193.2 ± 38	1074.3 ± 406
ICG 163	104 ± 7	113 ± 10	10 ± 3	733.9 ± 204	433 ± 83	1056.4 ± 194	91 ± 2	83 ± 9	22 ± 3	561.6 ± 73	338 ± 93	769.4 ± 233
ICG 2019	69 ± 7	105 ± 16	11 ± 3	510.7 ± 145	289.6 ± 82	615.1 ± 108	37 ± 25	49 ± 24	20 ± 4	384.1 ± 85	197.1 ± 73	474.3 ± 274
ICG 2106	108 ± 4	136 ± 11	10 ± 3	612.9 ± 132	329.7 ± 20	853.5 ± 83	75 ± 10	88 ± 18	18 ± 3	463.9 ± 40	239.7 ± 31	586.8 ± 318
ICG 3027	98 ± 11	130 ± 9	11 ± 3	752.9 ± 50	381.7 ± 41	892.4 ± 104	62 ± 2	82 ± 19	15 ± 2	613.9 ± 81	304.3 ± 49	718.2 ± 151
ICG 311	106 ± 9	135 ± 10	7 ± 2	638.5 ± 106	393.6 ± 71	676.2 ± 124	86 ± 8	101 ± 11	14 ± 1	562.8 ± 102	269 ± 20	543.1 ± 54
ICG 332	63 ± 1	83 ± 8	5 ± 2	658.8 ± 26	283.4 ± 34	985 ± 60	39 ± 5	47 ± 11	18 ± 2	457.5 ± 44	162.1 ± 37	726.5 ± 144
ICG 334	105 ± 9	159 ± 20	8 ± 1	884.7 ± 22	444.3 ± 29	813.5 ± 172	98 ± 7	143 ± 15	24 ± 2	692.3 ± 89	286.5 ± 32	553.5 ± 138
ICG 36	100 ± 13	112 ± 15	7 ± 2	778.8 ± 64	222.2 ± 35	737.3 ± 111	53 ± 7	67 ± 11	27 ± 3	528.2 ± 83	148.6 ± 13	463.2 ± 110
ICG 3992	73 ± 11	77 ± 20	10 ± 2	654.1 ± 130	317.7 ± 86	958.8 ± 107	47 ± 4	55 ± 9	23 ± 2	590.9 ± 73	224.1 ± 8	831.2 ± 86
ICG 4543	89 ± 11	126 ± 14	4 ± 5	485.4 ± 56	227.7 ± 19	785.4 ± 122	73 ± 6	87 ± 18	18 ± 1	385.3 ± 67	183.4 ± 52	578 ± 141
ICG 4598	98 ± 10	101 ± 16	7 ± 1	701.8 ± 143	356.4 ± 86	1050.8 ± 62	76 ± 7	89 ± 18	19 ± 1	497.8 ± 41	197 ± 9	803 ± 66
ICG 4684	87 ± 8	121 ± 25	10 ± 1	633.8 ± 95	347.9 ± 41	765.3 ± 97	71 ± 5	98 ± 18	19 ± 2	445.7 ± 53	246.8 ± 49	652.1 ± 87
ICG 4729	80 ± 2	94 ± 6	10 ± 2	393.3 ± 64	229 ± 17	711.7 ± 115	66 ± 7	80 ± 13	23 ± 1	329.7 ± 47	155.4 ± 28	530.2 ± 91
ICG 4750	89 ± 4	116 ± 5	9 ± 1	580.1 ± 27	338 ± 28	764.5 ± 86	75 ± 9	85 ± 10	27 ± 4	439.8 ± 46	186.7 ± 32	635 ± 109
ICG 4764	84 ± 7	125 ± 7	8 ± 2	727.3 ± 157	369.3 ± 68	839.8 ± 200	79 ± 3	108 ± 7	25 ± 3	549.6 ± 104	231.5 ± 24	643.7 ± 165
ICG 513	100 ± 14	138 ± 24	12 ± 4	861.2 ± 101	471.9 ± 98	815.3 ± 98	85 ± 6	110 ± 22	29 ± 2	688.8 ± 65	306 ± 12	696.7 ± 150
ICG 5195	134 ± 11	174 ± 12	11 ± 2	547.1 ± 21	302.3 ± 29	850.6 ± 27	98 ± 8	100 ± 6	25 ± 2	536 ± 51	264.4 ± 82	708.2 ± 185
ICG 532	90 ± 3	114 ± 7	10 ± 1	729.9 ± 123	298.2 ± 55	824.7 ± 145	71 ± 8	104 ± 2	18 ± 2	532.4 ± 96	213.1 ± 60	677.5 ± 110
ICG 5609	60 ± 6	98 ± 21	6 ± 2	524.9 ± 81	278.9 ± 14	750.7 ± 171	49 ± 10	58 ± 9	15 ± 3	411.5 ± 34	146.8 ± 17	556.8 ± 147
ICG 5663	144 ± 9	135 ± 6	17 ± 2	667.1 ± 78	313.9 ± 64	735.7 ± 2116	91 ± 13	85 ± 8	26 ± 3	541.8 ± 59	212.8 ± 51	569.4 ± 64
ICG 6263	76 ± 11	89 ± 14	8 ± 2	550.2 ± 41	152.1 ± 43	646.5 ± 131	53 ± 7	64 ± 15	21 ± 3	486.8 ± 44	86.1 ± 11	402 ± 117
ICG 6407	62 ± 4	79 ± 7	7 ± 3	522.4 ± 52	290.7 ± 17	833.9 ± 96	52 ± 3	67 ± 20	19 ± 2	500.9 ± 45	236.1 ± 34	741 ± 110
ICG 6654	89 ± 13	138 ± 8	9 ± 2	687.6 ± 94	342.7 ± 71	697.3 ± 121	70 ± 6	99 ± 6	18 ± 2	630.1 ± 77	265.3 ± 33	596 ± 125
ICG 6703	92 ± 7	116 ± 16	7 ± 2	546.3 ± 50	306 ± 44	932.7 ± 104	79 ± 5	100 ± 16	12 ± 2	377.6 ± 40	213 ± 23	517.2 ± 111
ICG 6813	138 ± 9	172 ± 14	11 ± 2	732.6 ± 68	377.9 ± 18	744.1 ± 429	125 ± 14	116 ± 3	25 ± 2	634.7 ± 56	343.3 ± 9	751.8 ± 98
ICG 6888	66 ± 9	80 ± 2	6 ± 2	540.8 ± 64	259.9 ± 81	830.6 ± 193	53 ± 5	67 ± 5	36 ± 2	417.5 ± 26	154.4 ± 22	657 ± 148
ICG 7181	69 ± 5	91 ± 15	10 ± 2	567.4 ± 51	310.9 ± 51	908.5 ± 89	62 ± 10	65 ± 5	22 ± 3	470.5 ± 77	220.3 ± 40	757 ± 105
ICG 721	106 ± 8	132 ± 21	11 ± 2	804.5 ± 75	409.4 ± 33	1064.5 ± 83	96 ± 10	123 ± 19	15 ± 2	638.1 ± 44	321.2 ± 46	954.4 ± 86
ICG 76	149 ± 22	162 ± 10	9 ± 2	933.6 ± 51	467.7 ± 69	807.4 ± 106	132 ± 9	131 ± 18	19 ± 3	727.2 ± 72	342.7 ± 41	618 ± 155
ICGIL 11102	141 ± 31	159 ± 29	8 ± 2	603.7 ± 62	282.9 ± 62	974.6 ± 139	119 ± 8	127 ± 21	23 ± 2	568.6 ± 56	248.3 ± 62	761.1 ± 177
ICGIL 11110	132 ± 13	151 ± 8	12 ± 2	833 ± 102	418.1 ± 55	848.4 ± 120	89 ± 9	103 ± 18	25 ± 2	578.8 ± 132	270.8 ± 29	567.8 ± 224
ICGIL 11114	70 ± 9	109 ± 22	5 ± 1	712 ± 110	423.5 ± 85	825.2 ± 158	65 ± 5	91 ± 19	21 ± 3	442.2 ± 135	264.8 ± 32	627.4 ± 182
ICGIL 11125	121 ± 4	132 ± 12	11 ± 1	724.4 ± 119	398.2 ± 56	728.5 ± 116	105 ± 10	108 ± 5	19 ± 2	674.3 ± 194	207.8 ± 61	594.2 ± 184
ICGIL 17108	135 ± 7	131 ± 5	15 ± 1	765.7 ± 98	180.7 ± 35	1022.2 ± 111	115 ± 6	89 ± 9	21 ± 2	582.3 ± 52	104.3 ± 23	808 ± 181
J 11	116 ± 8	127 ± 9	8 ± 1	497.4 ± 39	259.1 ± 39	697.3 ± 61	79 ± 8	87 ± 8	17 ± 2	443.7 ± 70	205.3 ± 13	493.5 ± 183
JL24	105 ± 9	140 ± 19	13 ± 2	565.8 ± 69	280.1 ± 37	720.6 ± 33	88 ± 3	126 ± 26	28 ± 2	488.5 ± 30	245 ± 52	539.2 ± 214

Means	102 ± 31	123 ± 31	9 ± 4	678.5 ± 204	316.7 ± 90	828.5 ± 279	80 ± 25	89 ± 27	22 ± 8	546.2 ± 164	230.4 ± 69	645.6 ± 215
LSD	15	21	5	131	79	145	12	20	9	107	56	140
